# Wellbeing and brain structure: A comprehensive phenotypic and genetic study of image‐derived phenotypes in the UK Biobank

**DOI:** 10.1002/hbm.25993

**Published:** 2022-06-29

**Authors:** Javad Jamshidi, Haeme R. P. Park, Arthur Montalto, Janice M. Fullerton, Justine M. Gatt

**Affiliations:** ^1^ Neuroscience Research Australia Sydney New South Wales Australia; ^2^ School of Psychology University of New South Wales Sydney New South Wales Australia; ^3^ School of Medical Sciences University of New South Wales Sydney New South Wales Australia

**Keywords:** cerebellum, cortex, magnetic resonance imaging, mediation, polygenic score, subcortical, wellbeing

## Abstract

Wellbeing, an important component of mental health, is influenced by genetic and environmental factors. Previous association studies between brain structure and wellbeing have typically focused on volumetric measures and employed small cohorts. Using the UK Biobank Resource, we explored the relationships between wellbeing and brain morphometrics (volume, thickness and surface area) at both phenotypic and genetic levels. The sample comprised 38,982 participants with neuroimaging and wellbeing phenotype data, of which 19,234 had genotypes from which wellbeing polygenic scores (PGS) were calculated. We examined the association of wellbeing phenotype and PGS with all brain regions (including cortical, subcortical, brainstem and cerebellar regions) using multiple linear models, including (1) basic neuroimaging covariates and (2) additional demographic factors that may synergistically impact wellbeing and its neural correlates. Genetic correlations between genomic variants influencing wellbeing and brain structure were also investigated. Small but significant associations between wellbeing and volumes of several cerebellar structures (*β* = 0.015–0.029, *P*_FDR_ = 0.007–3.8 × 10^−9^), brainstem, nucleus accumbens and caudate were found. Cortical associations with wellbeing included volume of right lateral occipital, thickness of bilateral lateral occipital and cuneus, and surface area of left superior parietal, supramarginal and pre‐/post‐central regions. Wellbeing‐PGS was associated with cerebellar volumes and supramarginal surface area. Small mediation effects of wellbeing phenotype and PGS on right VIIIb cerebellum were evident. No genetic correlation was found between wellbeing and brain morphometric measures. We provide a comprehensive overview of wellbeing‐related brain morphometric variation. Notably, small effect sizes reflect the multifaceted nature of this concept.

## Graphical abstract

Employing the UK Biobank Resource, we employ a quantitative multi‐item wellbeing‐index score to explore the relationships between wellbeing and brain morphometric features (including volume, thickness, and surface area, with extension to poorly studied regions of the cerebellum and brainstem) at both phenotypic and genetic levels. We show small but significant associations between the wellbeing‐index phenotype and volumes of several cerebellar structures, brainstem, nucleus accumbens and caudate, plus global measures of total grey matter volume and total surface area, and several associations with morphometric features of specific anatomical brain regions. Wellbeing‐PGS effects coincided with cerebellar volumes and supramarginal surface area—small mediation effects of wellbeing phenotype and PGS on right VIIIb cerebellum were evident, highlighting the cerebellum as a key wellbeing‐associated region.

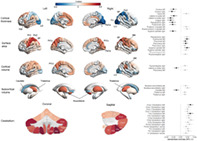

## 1 INTRODUCTION

A wealth of previous studies have focused on elucidating the neural correlates underlying mental illness phenotypes such as depression; however, the corresponding examination of the biological bases of wellbeing and underlying substrates that lead to positive mental health outcomes is in its infancy. Wellbeing is an essential aspect of mental health and is distinct from the absence of mental illness and psychopathology (Keyes, 2005), yet is negatively correlated with psychiatric or psychological traits such as depressive symptoms and neuroticism (Jamshidi et al., 2020; Jamshidi et al., 2022). Wellbeing is defined by two broad categories: subjective (also known as hedonic) wellbeing, which relates to positive affect and life satisfaction; and psychological (also known as eudaimonic) wellbeing which relates to attributes that enable realisation of human potential, such as mastery, autonomy and self‐worth (Deci & Ryan, 2006; Ryff et al., 2021). Previous magnetic resonance imaging (MRI) studies have mostly utilised voxel‐based morphometric methods to link grey matter volume (GMV) differences to wellbeing phenotypes, most with relatively small cohorts (*n* = ~50–1000). These studies have shown correlations between the volumes of anterior cingulate cortex (ACC; *n* = 106), and precuneus (*n* = 51) with subjective wellbeing (Matsunaga et al., 2016; Sato et al., 2015), right insula and psychological wellbeing (*n* = 70; Lewis et al., 2014), pons and composite wellbeing (indexing both subjective and psychological; *n* = 263; Gatt et al., 2018), hippocampus with subjective wellbeing (*n* = 724 twins and siblings; van't Ent et al., 2017) and left dorsolateral prefrontal cortex with social wellbeing (*n* = 294; Kong, Hu, Xue, et al., 2015c). Other structural studies of related constructs report associations between parahippocampal gyrus, precuneus and ventromedial prefrontal cortex with life satisfaction (*n* = 299; Kong, Ding, Yang, et al., 2015a), prefrontal and cingulate regions with quality of life (*n* = 156; Takeuchi et al., 2014), and a negative association between thickness of middle and superior frontal gyri and life satisfaction (*n* = 1031; Zhu et al., 2018). Nevertheless, there is little consistency across the reported findings, mainly due to limited sample sizes and diverse methodologies, including various operationalisations of the wellbeing construct. Thus, there is an unmet need for large population‐based neuroimaging studies using a data‐driven whole‐brain approach, to alleviate some of these issues.

Wellbeing is influenced by both environment and genetics with a moderate heritability (17–67%; Bartels, 2015). Genome‐wide association studies (GWAS) have mostly focused on subjective wellbeing, and have shown that wellbeing is highly polygenic, and estimate SNP‐heritability of ~4–9% (Baselmans et al., 2019; Jamshidi et al., 2022; Okbay et al., 2016). Thus, polygenic scores (PGS) can be used to quantitatively index the genetic signatures that underlie variability in wellbeing. Given that neuropsychiatric conditions and personality traits show relationships between PGS and brain structure (e.g. neuroticism [Opel et al., 2020], schizophrenia [Alnaes et al., 2019], ADHD [Alemany et al., 2019] and Autism [Khundrakpam et al., 2020]), and subjective wellbeing is negatively genetically correlated with these traits/disorders, relationships between wellbeing‐PGS and brain structure variability is reasonably expected. However, few studies have thus far examined associations between wellbeing‐PGS and brain morphometric measures. One study found a positive association between subjective wellbeing‐PGS and thickness of right superior temporal gyrus and volume of insula (*n* = 585; Song et al., 2019) while another found no evidence of wellbeing‐PGS mediation on hippocampal volume (*n* = 636 twins/siblings; van't Ent et al., 2017). PGS of anhedonia was also associated with reduced total GMV and thickness of para‐hippocampal, superior temporal gyrus and insula (*n* = 19,592; Zhu et al., 2021). Furthermore, imaging genetic studies have shown that brain structural variations are under polygenic influence (Grasby et al., 2020; Smith et al., 2021), but whether genetic variants that influence brain structure overlap with those that influence wellbeing is yet to be investigated.

Thus, the aim herein was to address these knowledge gaps and identify relationships between subjective wellbeing and brain morphometric measurements, examining both the wellbeing phenotype and wellbeing‐PGS effects. We took a whole‐brain approach examining brainstem, cerebellum, subcortical and cortical regions using Image‐derived phenotypes (IDPs) available in the UK Biobank. In addition to GMV, we also included cortical thickness (CT) and surface area (SA) to comprehensively examine structural morphometric associations. Given that CT and SA are posited to have distinct evolutionary origins and are influenced by different genes (Grasby et al., 2020), separate consideration from GMV is recommended (Winkler et al., 2010). For measuring wellbeing *phenotypically*, a factor score called the “wellbeing‐index” was employed, which quantifies subjective wellbeing measured across five domains (Jamshidi et al., 2022). For quantifying wellbeing *genetically*, a PGS was constructed in individuals with neuroimaging and genetic data, based on GWAS summary statistics of the “wellbeing‐index.” We then evaluated the association between phenotypic and genetic measures of wellbeing, with brain structure. Where main effects of both wellbeing phenotype and PGS on brain structure were observed, we employed mediation models to evaluate the process that underlies relationships between genotype, phenotype and brain variability. Finally, to determine whether genetic variants that influence brain structural variability covary with those that influence wellbeing, we evaluated genetic correlations between genetic signatures of brain structure and wellbeing.

## 2 MATERIALS AND METHODS

### 2.1 Participants

Participants were drawn from the UK Biobank Resource, a population‐based cohort with over 500,000 participants with physical and cognitive measures, self‐report questionnaires, biological samples and genetic data (Sudlow et al., 2015). Participants with MRI measures available in the first imaging visit (2014+) were included herein.

The sample selection workflow is provided in Figure S2. Participants who withdrew consent, reported neurological diseases or nervous system cancers [DataFields: 20001–20002], had missing wellbeing phenotype or covariate data, or had intracranial volume (ICV, described below) beyond three standard deviations (±3 SD) from the sample mean were excluded. After exclusions, 38,982 participants remained for phenotypic analysis. For PGS analysis, specific exclusions are described below.

All participants provided informed consent. The UK Biobank received approval from the National Health Service National Research Ethics Service (11/NW/0382). This project was approved by the UK Biobank Data Access Committee [projectID#58534], and The University of New South Wales Human Research Advisory Panel (HC200191).

### 2.2 Wellbeing phenotype

To measure wellbeing, we used a factor score named the “wellbeing‐index”, previously shown to be a good proxy for subjective wellbeing (Jamshidi et al., 2022), and is detailed in Supplementary Materials. In brief, the wellbeing‐index was generated by running a principal component analysis in SPSSv25 using items that index general happiness and satisfaction with family, friendship, health and financial situation [Data‐Fields: 4526, 4559, 4570, 4581 and 4548, respectively]; ascertained from the first imaging visit.

### 2.3 Brain image‐derived phenotypes

Image‐derived phenotypes (IDP) provided by the UK Biobank, derived from the processing of T1‐weighted images, were used herein. Details on image acquisition, pre‐processing and quality control are described elsewhere (https://biobank.ctsu.ox.ac.uk/crystal/crystal/docs/brain_mri.pdf; Miller et al., 2016). Briefly, regional GMV measures were generated by tissue‐type segmentation using FAST (FMRIB's Automated Segmentation Tool) [Category: 1101] (Zhang et al., 2001), and subcortical volumes were modelled using FIRST (FMRIB's Integrated Registration and Segmentation Tool) [Category: 1102] (Patenaude et al., 2011). Parcellations of cerebellum were derived from Diedrichsen Cerebellar Atlas (Diedrichsen et al., 2009). Mean CT, SA and GMV from 33 regions in each hemisphere, and global mean CT and total SA were derived from Desikan–Killiany Atlas[Category:192] (Desikan et al., 2006). In total, there were 245 IDPs included herein: total GMV, total white‐matter volume, brainstem volume, 28 cerebellar volumes, 14 subcortical volumes, 66 cortical volumes, 67 CTs and 67 SAs. ICV was calculated as the sum of total grey‐matter, white‐matter and ventricular cerebrospinal fluid volumes.

### 2.4 Polygenic scores and genetic correlation

The UK Biobank genetic data (March‐2018 release) was used herein. Detailed information on the cohort, genotyping, imputation and quality control is available elsewhere (Bycroft et al., 2018). Briefly, participants with non‐“British‐White” background [Data‐Field: 21000], mismatch between self‐reported and genotype‐derived sex, >10% genotype missingness or QC failure [Data‐Field: 22051], sex‐chromosome aneuploidy, those on the genomic analysis exclusion list [Data‐Field: 22010] and heterozygosity outliers [Data‐Field: 22027] were excluded. Furthermore, duplicates or relatives (kinship‐IBD >0.04) of individuals in the wellbeing discovery GWAS were excluded, to avoid bias due to discovery‐target sample overlap, leaving 19,986 participants (Figure S2).

We constructed wellbeing‐PGS based on the summary statistics of our previous wellbeing‐index GWAS (Jamshidi et al., 2022), using PRS‐CS software (Ge et al. 2019).

LD score regression (Bulik‐Sullivan et al., 2015) was used to estimate the genetic correlation between wellbeing‐index and specific brain IDPs that were found to be significantly associated with wellbeing‐index or wellbeing‐PGS. SNPs that specifically influence variability in brain IDPs were derived from publicly available summary statistics from GWAS of IDPs (Smith et al., 2021).

### 2.5 Statistical analyses

All IDPs, wellbeing scores and wellbeing‐PGS were rescaled into standardised z‐scores. Participants with wellbeing‐index or wellbeing‐PGS values beyond ±3 SD from the sample mean were excluded from analysis, and participants with region‐specific IDP values beyond ±3 SD were excluded case‐wise from that region‐specific IDP analysis (Figure S3A,B). We used separate multiple linear models to evaluate the association between IDPs and wellbeing‐index/wellbeing‐PGS, with wellbeing‐index/wellbeing‐PGS as the independent predictor, and IDP as the dependent outcome.

Two models were tested. In model 1, age (at MRI scan), age^2^, sex, ICV, assessment centre and scanner head position (x‐, y‐ and z‐axis) were included as covariates. Additionally, for PGS association analysis, genotype array and 10 principal components derived from genetic data were included as covariates in model 1. To test the robustness of the associations to other demographic features that may synergistically influence wellbeing or brain morphology, extra covariates were added to those basic covariates employed in model 1 to create model 2. Model 2 covariates were: education, ethnicity, Townsend deprivation index, smoking status, alcohol intake frequency and body mass index (BMI), plus model 1 covariates. Covariates are described in Supplementary Materials.

We divided analysis into the three sets of morphometric traits corresponding to volume, CT and SA. False Discovery Rate (FDR) was applied within each set for multiple testing using “p.adjust” function in R with a significance threshold of 0.0167 (*α* = 0.05/3) to account for three analysis sets. Finally, to explore potential sex effects on IDPs with significant association in the whole sample, we investigated the association between wellbeing and IDPs separately in males and females (using models 1 and 2, without sex as covariate).

### 2.6 Mediation analysis

For mediation analysis, only participants with scores for both wellbeing‐index and wellbeing‐PGS were included (*N* = 19,461). For regions that were significantly associated with both wellbeing‐index and wellbeing‐PGS in this sub‐sample, we tested whether brain morphometric measures mediate the association between PGS and wellbeing‐index. The R package *Lavaan v.0.6‐9* was used to test the mediation models, which included basic covariates from model 1.

## 3 RESULTS

### 3.1 Participant demographics

The phenotypic study comprised 38,982 participants, with a subset of 19,981 available for PGS analysis (Figure S2). The phenotypic sample was aged 45–82 years (mean ± SD = 63.86 ± 7.65) and 53% were female, with similar metrics for the PGS sample (age = 63.91 ± 7.49 years, 53% female; see Table 1).

**TABLE 1:** Participant demographic characteristics

Characteristic	Wellbeing‐index phenotype, *N* = 38,982^a^	Wellbeing‐index PGS, *N* = 19,987^a^
Age	63.86 (7.65)	63.91 (7.49)
Sex		
Female	20,776 (53%)	10,616 (53%)
Male	18,206 (47%)	9371 (47%)
Education		
No college degree	19,742 (51%)	10,535 (53%)
College degree	19,240 (49%)	9452 (47%)
Ethnicity		
Not White	1138 (2.9%)	0 (0%)^b^
White	37,844 (97%)	19,987 (100%)
Assessment centre		
Bristol	35 (<0.1%)	0 (0%)
Cheadle	23,251 (60%)	12,083 (60%)
Newcastle	9903 (25%)	5921 (30%)
Reading	5793 (15%)	1983 (9.9%)
Smoking status		
Never	24,527 (63%)	12,678 (63%)
Previous	13,163 (34%)	6667 (33%)
Current	1292 (3.3%)	642 (3.2%)
Alcohol intake frequency		
Daily or almost daily	6655 (17%)	3430 (17%)
Three or four times a week	11,117 (29%)	5807 (29%)
Once or twice a week	10,373 (27%)	5388 (27%)
One to three times a month	4496 (12%)	2237 (11%)
Special occasions only	3941 (10%)	1979 (9.9%)
Never	2400 (6.2%)	1146 (5.7%)
BMI (categorical)		
Underweight	273 (0.7%)	148 (0.7%)
Normal	15,855 (41%)	8171 (41%)
Overweight	15,993 (41%)	8147 (41%)
Obese	6861 (18%)	3521 (18%)
Townsend deprivation index	−1.92 (2.70)	−2.06 (2.67)
Wellbeing index	0.00 (1.00)	0.04 (0.96)

Abbreviations: BMI, body mass index; *n*, number; PGS, polygenic score; %, percentage.

^a^ Mean (SD) for continuous variables; *n* (%) for categorical variables.

^b^ Non‐white ethnicity was an exclusion criterion for inclusion in the PGS analysis.

The wellbeing‐index was correlated positively with age (Pearson's *r* = 0.11, *p* = 3.34 × 10^−102^) and negatively with Townsend deprivation index (Pearson's *r* = −0.10, *p* = 7.9 × 10^−88^). The mean wellbeing‐index was significantly different in groups relating to education level, ethnicity, assessment centre, smoking status, alcohol intake frequency and BMI (Figure S4).

### 3.2 Associations between wellbeing‐index phenotype and brain morphometric measures

**FIGURE 1:**
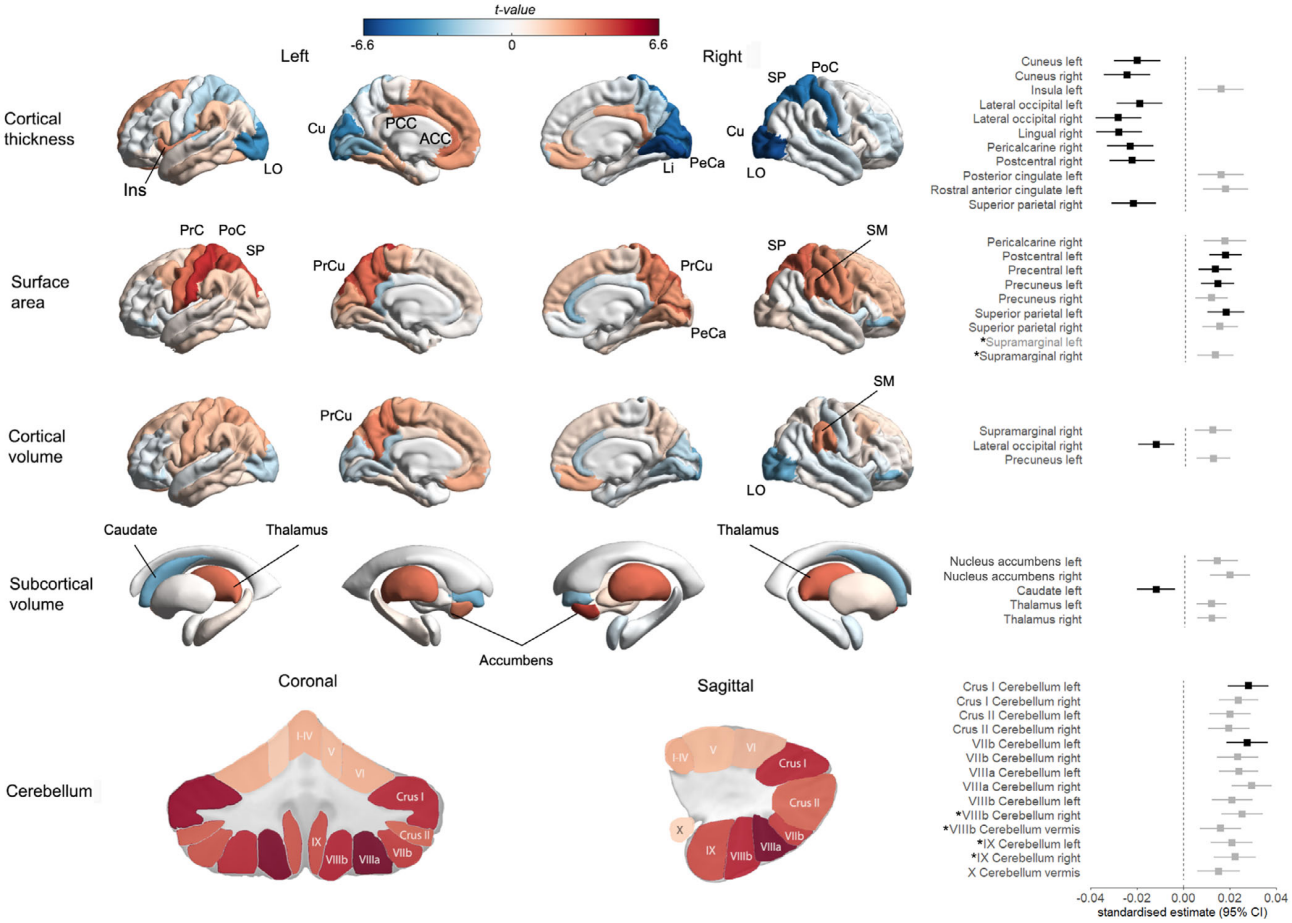
Brain maps of associations between wellbeing‐index and cortical thickness/surface area/volume, and subcortical and cerebellum volumes. The *t* value from the linear regressions are denoted on brain image renders, with negative associations indicated in blue hues and positive associations in red hues, where darker colours indicate stronger associations. The names of the IDPs that are significant after false discovery rate (FDR) correction for multiple tests (*P*_FDR_ <.016) are labelled on the brain images on the left, and are graphically presented on the right panel. The right panel shows the standardised estimates (β) with 95% confidence interval for FDR‐significant associations from model 1 (adjusted for age, age^2^, sex, ICV, assessment centre, and scanner x‐, y‐ and z‐axis positions). The IDPs that remained significant in model 2 (additionally adjusted for education, ethnicity, Townsend deprivation index, smoking status, alcohol intake frequency, and body mass index) are shown in black (effect sizes relate to model 1). The regions that were significantly associated with wellbeing‐PGS are specified with asterisks (*). The right supramarginal was only associated with the wellbeing‐PGS. Abbreviations: ACC, anterior cingulate cortex; cu, cuneus; Ins, insula; Li, lingual gyrus; LO, lateral occipital cortex; PCC, posterior cingulate cortex; PeCa, pericalcarine cortex; PoC, postcentral gyrus; PrC, precentral gyrus; PrCu, precuneus; SM, supramarginal gyrus; SP = superior parietal cortex.

Associations between wellbeing‐index and cerebellum, subcortical and cortical regions are presented in Figure 1 and Table 2.

**TABLE 2:** Associations between the wellbeing‐index phenotype and image‐derived phenotypes (IDPs)

Metric	IDP	Model 1			Model 2		
		Effect estimate (95%CI)	*p*_raw_	*P*_FDR_	Effect estimate (95%CI)	*p*_raw_	*P*_FDR_
Volume	Total GMV	0.0080 (0.0047–0.0113)	1.57E‐06	1.96E‐05	0.0029 (‐0.0003–0.0062)	0.0778	0.2540
	Brainstem	0.0157 (0.0078–0.0236)	9.85E‐05	7.29E‐04	0.0171 (0.0091–0.0251)	2.75E‐05	0.0031
	Accumbens right	0.0195 (0.0108–0.0281)	1.12E‐05	9.88E‐05	0.0144 (0.0057–0.0232)	0.0012	0.0177
	Accumbens left	0.0141 (0.0055–0.0228)	0.0014	0.0070	0.0100 (0.0012–0.0187)	0.0256	0.1779
	Thalamus right	0.0117 (0.0055–0.0178)	2.26E‐04	0.0016	0.0074 (0.0011–0.0136)	0.0207	0.1703
	Thalamus left	0.0115 (0.0051–0.0179)	4.26E‐04	0.0028	0.0070 (0.0005–0.0134)	0.0341	0.1961
	Caudate left	‐0.0124 (−0.0206−‐0.0043)	0.0026	0.0127	‐0.0148 (‐0.023−0.0066)	4.02E‐04	0.0100
	VIIIa Cerebellum right	0.0293 (0.0206–0.0379)	3.41E‐11	3.79E‐09	0.0135 (0.0049–0.0220)	0.0020	0.0225
	Crus I Cerebellum left	0.0279 (0.0192–0.0365)	3.35E‐10	1.86E‐08	0.0177 (0.0090–0.0264)	7.13E‐05	0.0040
	VIIb Cerebellum left	0.0274 (0.0185–0.0363)	1.44E‐09	5.34E‐08	0.0156 (0.0067–0.0245)	5.74E‐04	0.0106
	VIIIb Cerebellum right	0.0251 (0.0162–0.034)	3.02E‐08	8.02E‐07	0.0092 (0.0004–0.0180)	0.0411	0.2076
	Crus I Cerebellum right	0.0237 (0.0153–0.0321)	3.61E‐08	8.02E‐07	0.0139 (0.0054–0.0224)	0.0013	0.0177
	VIIIa Cerebellum left	0.0237 (0.0151–0.0323)	6.04E‐08	1.12E‐06	0.0095 (0.0010–0.0180)	0.0281	0.1838
	VIIb Cerebellum right	0.0233 (0.0144–0.0321)	2.71E‐07	4.30E‐06	0.0117 (0.0028–0.0205)	0.0100	0.0929
	IX Cerebellum right	0.0221 (0.0131–0.0311)	1.59E‐06	1.96E‐05	0.0089 (‐0.0001–0.0179)	0.0515	0.2183
	VIIIb Cerebellum left	0.0208 (0.0121–0.0296)	3.22E‐06	3.57E‐05	0.0074 (‐0.0013–0.0161)	0.0943	0.2755
	IX Cerebellum left	0.0208 (0.0118–0.0297)	6.01E‐06	6.06E‐05	0.0088 (‐0.0002–0.0177)	0.0553	0.2191
	Crus II Cerebellum left	0.0201 (0.0111–0.029)	1.16E‐05	9.88E‐05	0.0091 (0.0001–0.0181)	0.0468	0.2164
	Crus II Cerebellum right	0.0194 (0.0105–0.0284)	2.11E‐05	1.67E‐04	0.0092 (0.0003–0.0182)	0.0433	0.2090
	VIIIb Cerebellum vermis	0.0159 (0.0069–0.0249)	5.21E‐04	0.0032	0.0037 (‐0.0053–0.0127)	0.4173	0.6504
	X Cerebellum vermis	0.0150 (0.0059–0.0242)	0.0013	0.0070	0.0098 (0.0006–0.0191)	0.0376	0.1987
	Precuneus left	0.0120 (0.0048–0.0192)	0.0011	0.0065	0.0096 (0.0023–0.0169)	0.0100	0.0929
	Supramarginal right	0.0119 (0.004–0.0198)	0.0031	0.0144	0.0094 (0.0014–0.0174)	0.0217	0.1703
	Lateral occipital right	‐0.0127 (‐0.0204−‐0.0049)	0.0014	0.0070	‐0.0154 (‐0.0232−‐0.0075)	1.22E‐04	0.0045
	Parsorbitalis right*	‐0.0125 (‐0.0209−‐0.004)	0.0038	0.0168	‐0.0153 (‐0.0238−‐0.0067)	4.52E‐04	0.0100
Thickness	Rostral anterior cingulate left	0.0172 (0.0074–0.027)	5.65E‐04	0.0042	0.0121 (0.0023–0.0220)	0.0160	0.0827
	Posterior cingulate left	0.0153 (0.0054–0.0252)	0.0025	0.0156	0.0128 (0.0028–0.0228)	0.0122	0.0681
	Insula left	0.0152 (0.0053–0.0252)	0.0026	0.0156	0.0088 (‐0.0012–0.0188)	0.0838	0.2674
	Lateral occipital right	‐0.0289 (‐0.0387−‐0.0191)	8.12E‐09	3.99E‐07	‐0.0283 (‐0.0382−‐0.0184)	2.15E‐08	1.44E‐06
	Lingual right	‐0.0287 (‐0.0386−‐0.0189)	1.19E‐08	3.99E‐07	‐0.0245 (‐0.0345−‐0.0145)	1.48E‐06	4.96E‐05
	Cuneus right	‐0.0252 (‐0.0352−‐0.0152)	7.72E‐07	1.72E‐05	‐0.0223 (‐0.0324−‐0.0122)	1.46E‐05	1.95E‐04
	Pericalcarine right	‐0.0238 (‐0.0338−‐0.0138)	2.81E‐06	4.71E‐05	‐0.0177 (‐0.0277−‐0.0076)	5.61E‐04	0.0062
	Postcentral right	‐0.0229 (‐0.0326−‐0.0132)	3.57E‐06	4.79E‐05	‐0.0229 (‐0.0327 −‐0.0132)	4.32E‐06	9.66E‐05
	Superior parietal right	‐0.0224 (‐0.0319−‐0.0128)	4.92E‐06	5.49E‐05	‐0.0217 (‐0.0313−‐0.012)	1.10E‐05	1.85E‐04
	Cuneus left	‐0.0208 (‐0.0307−‐0.0108)	4.49E‐05	4.29E‐04	‐0.0168 (‐0.0268−‐0.0067)	0.0011	0.0088
	Lateral occipital left	‐0.0198 (‐0.0296−‐0.0099)	8.62E‐05	7.22E‐04	‐0.0173 (‐0.0272−‐0.0074)	6.47E‐04	0.0062
Surface area	Postcentral left	0.0172 (0.0102–0.0243)	1.50E‐06	1.00E‐04	0.0163 (0.0093–0.0234)	6.10E‐06	4.09E‐04
	Superior parietal left	0.0174 (0.0095–0.0254)	1.81E‐05	6.06E‐04	0.0152 (0.0072–0.0232)	2.12E‐04	0.0071
	Pericalcarine right	0.0170 (0.0077–0.0263)	3.44E‐04	0.0034	0.0142 (0.0048–0.0236)	0.0030	0.0175
	Superior parietal right	0.0149 (0.0072–0.0226)	1.62E‐04	0.0024	0.0124 (0.0046–0.0202)	0.0019	0.0175
	Precuneus left	0.0138 (0.0066–0.0211)	1.78E‐04	0.0024	0.0127 (0.0054–0.0200)	6.90E‐04	0.0116
	Supramarginal right	0.0129 (0.0052–0.0207)	0.0010	0.0087	0.0121 (0.0043–0.0199)	0.0024	0.0175
	Precentral left	0.0128 (0.0058–0.0198)	3.60E‐04	0.0034	0.0130 (0.0059–0.0201)	3.48E‐04	0.0078
	Precuneus right	0.0112 (0.0042–0.0183)	0.0018	0.0138	0.0108 (0.0036–0.0179)	0.0031	0.0175
	Total SA	0.0076 (0.0036–0.0115)	1.62E‐04	0.0024	0.0062 (0.0022–0.0102)	0.0022	0.0175

*Note*: Associations that exceed FDR‐corrected significance threshold (*P*_FDR_ <.016; adjusted across volume, thickness and area morphometric sets) in model 1 or model 2 are listed, alongside their effect estimate (*β*), 95% confidence interval of effect estimate (95%CI), raw *p* value (*p*_raw_), FDR‐corrected *p* value (*P*_FDR_) for both model 1 and 2. Model 1 included age (at MRI scan), age^2^, sex, ICV, assessment centre, and scanner x‐, y‐ and z‐axis positions. Model 2 included model 1 covariates as well as education, ethnicity, Townsend deprivation index, smoking status, alcohol intake frequency, and body mass index (BMI). *Parsorbitalis right association was only significant (*P*_FDR_ <0.016) in model 2.

Abbreviations: IDP, image‐derived phenotype; GMV, grey matter volume; SA, surface area.

### 3.3 Cerebellum, brainstem and subcortical volumes

In model 1, 14 out of 28 segmented volumes of the cerebellum were positively associated with wellbeing‐index (*β* = 0.015–0.029, *P*_FDR_ = 0.007–3.8 × 10^−9^; Table 2), with the strongest association for the right VIIIa region. There was a positive association between wellbeing‐index and volume of the brainstem. For subcortical regions, higher wellbeing‐index was significantly associated with increased bilateral volume of nucleus accumbens and thalamus, and reduced volume of left caudate. In addition, there was a small but significant association between higher wellbeing‐index and increased total GMV.

In model 2, the directions of effect remained the same for all regions, but with a slightly larger effect size for the brainstem and caudate and smaller effect size for the cerebellum. Associations remained significant for brainstem, and left lateralised regions of caudate, cerebellum crus I and cerebellum VIIb.

### 3.4 Cortical volumes

In model 1, higher wellbeing‐index was associated with increased volume of the right supramarginal gyrus (SMG), and left precuneus, and reduced volume of the right lateral occipital region. In model 2, only the association with the right lateral occipital region remained significant. Furthermore, the nominal association of wellbeing‐index with right pars orbitalis of the inferior frontal gyrus in model 1 (*β* = −0.012, *P*_FDR_ = 0.0168) became FDR‐significant in model 2 (*β* = −0.015, *P*_FDR_ = 0.010).

### 3.5 Cortical thickness

In model 1, higher wellbeing‐index was significantly associated with *decreased* mean CT bilaterally in the cuneus and lateral occipital regions, the right lateralised lingual, pericalcarine, postcentral and superior parietal regions, and *increased* mean thickness in left insula, posterior cingulate and rostral anterior cingulate regions.

In model 2, associations were generally attenuated but remained significant for bilateral cuneus and lateral occipital, and right lateralised effects in the lingual, pericalcarine, postcentral and superior parietal regions.

### 3.6 Surface area

In model 1, higher wellbeing‐index was significantly associated with increased total SA and regional SA increases in left postcentral, superior parietal and precentral regions, and right pericalcarine, superior parietal and supramarginal regions as well as precuneus bilaterally.

In model 2, the left lateralised associations remained significant for postcentral, superior parietal, precentral and precuneus regions.

### 3.7 Associations between wellbeing‐PGS and brain morphometric measures

In model 1, significant associations of higher wellbeing‐PGS were observed with increased volume of four regions of cerebellum including right VIIIb (*β* = 0.027, *P*_FDR_ = 0.001), vermis VIIIb (*β* = 0.022, *P*_FDR_ = 0.016), right IX (*β* = 0.027, *P*_FDR_ = 0.001) and left IX (*β* = 0.027, *P*_FDR_ = 0.001). A nominal association between PGS and total GMV did not survive FDR‐correction for the three morphometric measures (*β* = 0.007, *P*_FDR_ = 0.019).

In model 1, wellbeing‐PGS was not significantly associated with CT measures in any region. However, wellbeing‐PGS was significantly associated with increased SA of the supramarginal region bilaterally (left: *β* = 0.023, *P*_FDR_ = 0.002; right: *β* = 0.021, *P*_FDR_ = 0.005).

In model 2, the association of supramarginal SA and wellbeing‐PGS remained significant (left: *β* = 0.023, *P*_FDR_ = 0.003; right: *β* = 0.021, *P*_FDR_ = 0.006).

### 3.8 Mediation analysis

**FIGURE 2:**
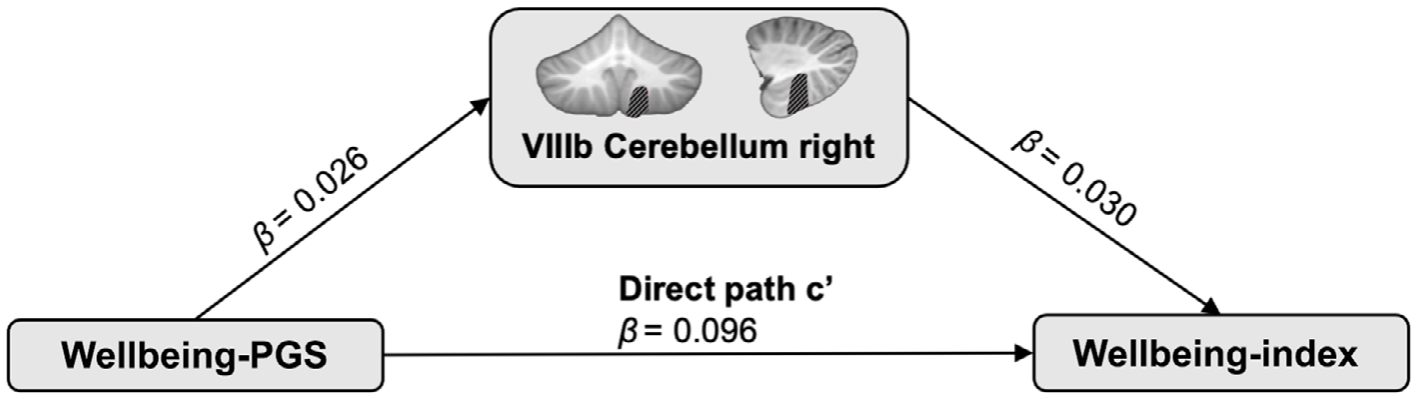
Path diagram of the mediation model of wellbeing‐PGS, right VIIIb cerebellum and wellbeing‐index. The model assesses the mediation of the volume of right VIIIb cerebellum (shown in the central panel by hatched diagonal shading with respect to the Diedrichsen cerebellar atlas) in the association between wellbeing‐PGS and wellbeing‐index. The standardised effect sizes (β) are presented for each path. *p* value is <0.001 for all paths.

Association analyses were repeated in the smaller mediation sample (*n* = 19,461), with main effects summarised in Figure S5. Only right VIIIb cerebellum had significant (*P*_FDR_ <0.016) associations with both wellbeing‐index and wellbeing‐PGS in the smaller sample. The mediation model showed that right VIIIb cerebellum partially mediates ~0.8% of the association between wellbeing‐index phenotype and wellbeing‐PGS (total effect: *β* = 0.097, 95% CI = 0.083–0.110, *p* < .001; total indirect effect: *β* = 0.0008, 95% CI = 0.0002–0.001, *p* = .005). Coefficients for individual paths are presented in Figure 2.

### 3.9 Genetic correlation between wellbeing‐index and brain morphometric measures

Of the 45 brain morphometric variables that were significantly associated with either the wellbeing‐index or wellbeing‐PGS, none had a significant *genetic* correlation (*r*_*g*_) with wellbeing‐index after correction for multiple testing, indicating little overlap between the main genetic drivers of brain structural variability with those that influence wellbeing. However, *nominally* significant (*p* < .05) genetic correlations with wellbeing‐index phenotype were observed for thickness of right cuneus (*r*_*g*_ = −0.110, *p* = .028), left rostral anterior cingulate (*r*_*g*_ = −0.112, *p* = .034) and left insula (*r*_*g*_ = −0.099, *p* = .037).

### 3.10 Gender‐stratified associations

The result of sex‐stratified association analysis between wellbeing‐index and IDPs is presented in Figure S6. The patterns of associations were mostly similar in males and females. However, for some regions the association was stronger in females (e.g. volume of left caudate and thalamus), and for some regions stronger in males (e.g. thickness of right cuneus and pericalcarine). Thickness of right lateral occipital was the only IDP significantly associated with wellbeing in both males and females in model 2. Some associations were not significant in either male or female subsets, which may be due to smaller sample sizes.

## 4 DISCUSSION

We investigated the phenotypic and polygenic associations between a wellbeing‐index score and brain morphometric measures, exploring possible overlaps in the genetic contributors to cortical and subcortical variability and wellbeing. Given the dearth of well‐powered neuroimaging studies of wellbeing in the literature, most of the findings reported herein were novel. Furthermore, as most previous studies focused on relationships between wellbeing and volumetric morphology, the present study—which also included modalities of cortical thickness (CT) and surface area (SA), plus extension to poorly studied regions of the cerebellum and brainstem—provides further novel insights into the relationship between wellbeing and brain structure.

Global measures of total GMV and total SA were positively correlated with the wellbeing‐index phenotype, but global CT was not associated. Several associations between wellbeing‐index and anatomical brain regions were also observed. In general, higher wellbeing was associated with increased regional SA, whereas associations with regional thickness tended to be negative, suggesting that the factors that influence or are impacted by wellbeing are differentially associated with these unique but interrelated measures of brain morphometry (Grasby et al., 2020; Winkler et al., 2010). On the whole, despite small effect sizes (standardised *β* = 0.008–0.029), after controlling for additional confounders (in model 2) that might uniquely influence both wellbeing and brain morphometry (such as BMI, socioeconomic status and alcohol use [Hamer & Batty, 2019; Spear, 2018; Yaple & Yu, 2020]), the directions of effect remained the same for associations found in model 1. However, not all of the associations remained significant in model 2, suggesting that these confounders may mediate/moderate some associations. Interestingly, both wellbeing phenotype and wellbeing‐PGS were associated with volume of four cerebellar regions and supramarginal SA. The lack of genetic correlation between wellbeing‐index and brain morphometric measures may indicate lack of effect (suggesting non‐genetic factors are the main drivers of wellbeing‐related IDP variation) or the covariance between genetic variations that influence brain morphometric variability (generally) and those that influence wellbeing (specifically) is too small to detect in the present sample size.

### 4.1 Cerebellum volume

One of the most striking findings from the present study was an association between multiple regions of the cerebellum (specifically the posterior lobe) and wellbeing, where those with higher wellbeing‐index scores displayed greater cerebellar volume. Furthermore, the significant association of wellbeing‐PGS with cerebellar regions suggests that genetic factors are integral to cerebellum–wellbeing associations, somewhat consistent with relatively high heritability of cerebellar structures (Zhao et al., 2019). Interestingly, our earlier study, in which we initially defined and validated the wellbeing‐index, found that wellbeing‐related genes identified via GWAS were enriched in genes that are differentially expressed in the cerebellum (Jamshidi et al., 2022). Thus, the present neuroanatomical associations further support the cerebellum as a conduit of wellbeing phenotypic and genetic associations. However, the weak mediation effect of VIIIb cerebellum volume in wellbeing‐PGS association and lack of genetic correlation between wellbeing and cerebellar volumes indicates that this association might be due to gene–environment correlations/interactions rather than direct genetic effects.

Neuroimaging studies have shown that in addition to motor functions, the cerebellum is involved in processing emotions (Baumann & Mattingley, 2012). The right posterior cerebellum has shown peak functional activation in the imagination of happiness (Costa et al., 2019). Furthermore, cerebral lesions can disrupt affective processes (Pierce & Peron, 2020). For example, patients with cerebellar lesions can display atypical and impaired responses to positive and negative stimuli (Clausi et al., 2015; Clausi et al., 2019; Turner et al., 2007), suggesting that the cerebellum plays a role in the subjective evaluation of emotion (Adamaszek et al., 2017). Decreased volume or damage to the cerebellum has also been associated with increased depression and anxiety symptoms in healthy individuals (Schutter et al., 2012), bipolar‐related mood disorders (Lupo et al., 2019), and major depressive disorder (Abe et al., 2010; Frodl et al., 2008; Peng et al., 2011). Considering the importance of emotion processing in wellbeing, our finding of a cerebellar–wellbeing relationship further consolidates the role of this structure in the wellbeing phenotype.

### 4.2 Brainstem volume

We report that increased brainstem volume was associated with improved wellbeing‐index score. In addition to basic sleep and respiratory functions, the brainstem is involved in neuromodulatory systems, impacting higher‐level emotional and cognitive functioning (Avery & Krichmar, 2017; Benarroch, 2018; Venkatraman et al., 2017). The brainstem has been implicated in association with wellbeing in two previous studies: an 8 week mindfulness‐based stress reduction intervention (*n* = 14) in which improved psychological wellbeing was correlated with increased grey matter density in two clusters within the brainstem (Singleton et al., 2014); and a cross‐sectional study (*n* = 263 twins) that employed a composite wellbeing scale which negatively correlated with volume of the pontine nuclei (Gatt et al., 2018). While it is possible that these conflicting effects are due to smaller sample sizes or different wellbeing measures, further analysis of specific substructures within the brainstem in association with wellbeing is warranted.

### 4.3 Subcortical volumes

Wellbeing‐index was positively associated with nucleus accumbens and thalamus volumes and negatively associated with caudate volume. Nucleus accumbens and caudate, as parts of the basal ganglia, are crucial structures within the reward circuit (Haber & Knutson, 2010; Pierce & Peron, 2020), particularly in the processing of pleasure, and emotional and motivational processes (Salgado & Kaplitt, 2015). The thalamus is involved in emotion regulation via limbic‐thalamo‐cortical projections (Ward, 2013), and previous research has reported positive correlations between optimism and left thalamic (Yang et al., 2013) and left nucleus accumbens volume in young adults (Dolcos et al., 2016). Similarly, anhedonia and lifetime major depressive disorder (MDD; negatively associated with wellbeing) are associated with *smaller* thalamus and nucleus accumbens volumes (Ancelin et al., 2019; Zhu et al., 2021). In terms of the caudate, a negative association between left caudate volume and subjective wellbeing (*n* = 49, aged 12 years; Boyes et al., 2022) is supported by the current study.

### 4.4 Cortical associations

Prior structural morphometric studies have almost exclusively focused on associations between measures of GMV and wellbeing: we found no studies that explored relationships to cortical SA, and only one study that examined associations with life satisfaction explored CT measures (Zhu et al., 2018).

Frontal lobe regions such as prefrontal cortex, superior frontal gyrus, middle frontal gyrus and anterior cingulate cortex have been implicated by several studies of wellbeing (or related traits, e.g. life satisfaction; Kong et al., 2020; Kong, Ding, Yang, et al., 2015a; Kong, Hu, Xue, et al., 2015c; Matsunaga et al., 2016; Takeuchi et al., 2014; Zhu et al., 2018). Although we found little support for volumetric variations in the frontal lobe with wellbeing, we found a positive association between wellbeing‐index and the *thickness* of left rostral anterior cingulate. The anterior cingulate is involved in various functions such as emotion, self‐monitoring, motivation and cognitive control (Etkin et al., 2011; Tang et al., 2019), and is a structure commonly associated with wellbeing (King, 2019), although directions of effect and primary substructures are not always consistent. The rostral anterior cingulate activity is also implicated in optimism (Erthal et al., 2021).

Our finding of a positive correlation between left precentral gyrus SA and wellbeing‐index is somewhat novel. The precentral gyrus contains the primary motor cortex and a portion of the supplementary motor area (SMA), mainly responsible for planning and controlling voluntary movements (Banker & Tadi, 2022) and may play a role in emotion‐action interactions (Blakemore & Vuilleumier, 2017). Somewhat consistent with our SA finding, greater *volume* in the left SMA is associated with higher subjective wellbeing (Wang et al., 2020). Indeed, engagement in physical activity can impact subjective wellbeing (Wiese et al., 2017). The postcentral gyrus, in which we identified wellbeing‐associated decreased thickness and increased SA, processes proprioception (i.e. sensations of touch, temperature, pain; DiGuiseppi & Tadi, 2022), and is involved in generation/regulation of emotion and internal awareness of emotional state (Kropf et al., 2019). Brain activity in postcentral gyrus has been associated with cognitive wellbeing (Kong, Hu, Wang, et al., 2015b), and its volume has been related to anxious‐depression (Peng et al., 2019).

Our study also highlights several wellbeing‐associated regions in the parietal lobe, including increased posterior cingulate cortex and insula thickness, and increased volume and SA of precuneus and supramarginal gyrus. The posterior cingulate is involved in emotional processing and episodic memory (Leech & Smallwood, 2019), and can be activated by external emotional stimuli (Maddock et al. 2003), and self‐reflection (Northoff et al., 2006). Higher resilience scores have previously been associated with increased thickness of the left posterior cingulate (Shikimoto et al., 2021), and several functional studies have illuminated relationships between wellbeing and posterior cingulate activity (Kong, Hu, Wang, et al., 2015b; Luo et al., 2014; Luo et al., 2016; Ren et al., 2019). However, associations between precuneus volume and life satisfaction (Kong, Ding, Yang, et al., 2015a) and subjective wellbeing (Sato et al., 2015) have shown different lateralisation and directions of effect.

The significant association of both the wellbeing‐index phenotype and wellbeing‐PGS with supramarginal gyrus SA suggests that this association might have a stronger genetic component. However, we did not detect genetic correlations between supramarginal gyrus SA and wellbeing‐index, suggesting the involvement of other mediators/moderators and the possibility of gene–environment correlation/interaction in the association. The supramarginal gyrus has roles in both cognitive and emotional functions such as phonological processing (Hartwigsen et al., 2010), emotion regulation (Wadden et al., 2018), and emotion recognition (Wada et al., 2021). Supramarginal structural variations might affect wellbeing by influencing the recognition of emotions in others and potentially regulating empathic behaviours (Silani et al., 2013).

We also report a positive correlation between left insula thickness and wellbeing. Although the insular cortex has heterogenous functions (e.g. sensorimotor, socio‐emotional and cognitive processing), it has an important role in self‐generated thought and processing subjective feelings (Fox et al., 2018), which highlights its possible role in subjective wellbeing. Furthermore, earlier studies have shown that insula volume is positively associated with psychological wellbeing (Lewis et al., 2014) and health‐related quality of life (Hahm et al., 2019), and negatively associated with lifetime MDD (Ancelin et al., 2019).

Unexpectedly, we found several significant associations in the occipital lobe, including pericalcarine, cuneus, lingual and lateral occipital cortex. The occipital lobe is mainly involved in visual information processing, such as visuospatial processing and object and face recognition (Rehman & al Khalili, 2022). The only relevant report regarding the occipital lobe is the association between wellbeing and resting‐state regional activity in the right lingual gyrus (Kong, Hu, Wang, et al., 2015b). However, decreased activity within the occipital lobe and lingual gyrus has been reported in MDD patients (Zhong et al., 2016). Furthermore, people with lifetime depression had larger volumes in the pericalcarine and lingual gyrus (Ancelin et al., 2019; Du et al., 2014). Structural variation in the occipital lobe might influence wellbeing via alterations in perception and interpretation of visual information (specifically those with emotional valence such as facial expressions), or even the ability to use visual imagery, which is practiced in some meditative techniques.

Interestingly, we observed a significant association of decreased CT and increased SA in the right pericalcarine with higher wellbeing. This opposite direction of CT and SA effects might mask detection with volumetric measures, highlighting the importance of examining CT and SA independently to GM volume, as previously recommended (Winkler et al., 2010).

### 4.5 Limitations

Our study inherits the limitations and biases of the UK Biobank sample. Most participants (including all participants in the PGS subsample) are of “British‐White” ethnic background. Participants might be healthier, better educated, or with a higher socioeconomic status than the general population (Davis et al., 2020). While most previous studies on the neural correlates of wellbeing have recruited young people, the older age of UK Biobank participants (i.e. >45 years) might influence the associations reported herein, as brain structure (Hogstrom et al., 2013) and wellbeing (Steptoe et al., 2015) are both subject to age‐related temporal changes. Furthermore, the statistical power for detecting PGS‐brain associations was smaller than phenotype–brain associations due to the smaller PGS sample. To maximise power, we did not limit the phenotypic sample (~39 k) to participants with PGS data (~20 k) but note that some phenotype–brain associations lost statistical significance in the smaller (mediation) sample. While the direction and size of effects were consistent, this highlights the need for sample sizes upwards of ~20 k participants for replication (at least within general populations examining cross‐sectional associations), and future novel discovery. PGS–brain associations may become more informative with future discovery of wellbeing‐associated genetic variants that explain a larger proportion of phenotypic variance. Furthermore, our whole‐brain analysis was limited to the regional‐specific parcellations defined by the neuroanatomical atlases used to derive the IDPs, hence associations that span parcellation boundaries, or relate to substructures within IDPs (e.g. anterior and posterior insula) may have not been detected. Moreover, we provide inference of potential functional impact of observed structural changes under the assumption that a brain structure–function relationship exists, but note that structural morphological differences may not result in behavioural change that is implied by spatial mapping of a functional parameter (Roskies, 2022). Finally, we examined associations with one index measure of subjective wellbeing, and did not separately analyse individual facets of wellbeing, that may reveal differential effects. Importantly, we did not examine measure of psychological wellbeing, therefore our results are limited to subjective wellbeing. Future studies could explore differential associations with each facet of subjective (e.g. positive affect, life satisfaction) or psychological (e.g. mastery, life purpose) wellbeing.

## 5 CONCLUSION

We provide a comprehensive roadmap of the phenotypic and genetic associations between a subjective wellbeing‐index and brain morphological variations. Evidence for previously identified regions was consolidated (e.g. precuneus, cingulate cortex and insula), and novel regions were implicated (e.g. visual and somatosensory cortex). While effect sizes were small, we find a broad pattern of structural variations underlying differences in wellbeing, which reflects the multifaceted nature of this concept.

## AUTHOR CONTRIBUTIONS

The study was conceptualised by Javad Jamshidi, Janice M. Fullerton and Justine M. Gatt. Analysis methodology was developed by Javad Jamshidi, Justine M. Gatt and Janice M. Fullerton, and administrative efforts and resources were provided by Janice M. Fullerton and Justine M. Gatt. Formal analysis was conducted by Javad Jamshidi with supervision by Janice M. Fullerton and Justine M. Gatt. Assistance in the mediation analyses, graphical representation of association results on brain maps, and interpretation and presentation of findings were contributed to by Haeme R. P. Park and Arthur Montalto. The original draft was written by Javad Jamshidi, with review & editing by Javad Jamshidi, Haeme R. P. Park, Arthur Montalto, Justine M. Gatt and Janice M. Fullerton, who all approved the final manuscript. Funding relevant to the current work was acquired by Javad Jamshidi (UNSW Scientia PhD scholarship) with the support of Justine M. Gatt and Janice M. Fullerton, and also was supported by grants acquired by Justine M. Gatt and Janice M. Fullerton.

## CONFLICT OF INTEREST

Justine M. Gatt is a stockholder in MAP Biotech Pty Ltd. There are no other conflicts of interest to report, nor competing financial interests in relation to the work described.

## Supporting information


**APPENDIX S1** Supporting Information

## Data Availability

The UKB Data described in this manuscript, originally cited in Sudlow et al. (2015) and Miller et al. (2016), is available to all researchers and can be accessed upon approval of the UK Biobank (https://www.ukbiobank.ac.uk/enable-your-research/apply-for-access). Analytic codes used in this study are stored in the UNSW data archive at https://www.dataarchive.unsw.edu.au, under Research Data Management Plan reference number H0237934, and are available on request.
