# Supplementary material for: Wellbeing and brain structure: A comprehensive phenotypic and genetic study of image‐derived phenotypes in the UK Biobank
Source: Hum Brain Mapp. 2022 Jun 29;43(17):5180–93. doi: 10.1002/hbm.25993 (PMC9812238; doi:10.1002/hbm.25993)
Supplement: Supplementary file 1 — APPENDIX S1 Supporting Information [file HBM-43-5180-s001.docx]

**Supplementary Information**

**Wellbeing and brain structure; a comprehensive phenotypic and genetic study** **of image-derived phenotypes in the UK Biobank**

Javad Jamshidi^1,2^, Haeme R P Park^1,2^, Arthur Montalto^1,2^, Janice M Fullerton^1,3^* & Justine M Gatt^1,2^*

*joint senior authors

1. Neuroscience Research Australia, Sydney, NSW, Australia

2. School of Psychology, University of New South Wales, Sydney, NSW, Australia

3. School of Medical Sciences, University of New South Wales, Sydney, NSW, Australia

Table of Contents

[1. Supplementary methods 1](#_Toc103528737)

[1.1. Derivation of the Wellbeing-index 1](#_Toc103528738)

[**Figure S1. Scree plot and distribution of the wellbeing-index score.** A) The scree plot from the principal component analysis (PCA) and, B) Distribution of the wellbeing-index in the sample. 2](#_Toc103528739)

[1.2. Sample selection 1](#_Toc103528740)

[**Figure S2. The sample selection workflow** 1](#_Toc103528741)

[1.3. Covariates 1](#_Toc103528742)

[**Figure S3A. The number of participants with extreme values for each IDP in wellbeing-index phenotype analysis.** 2](#_Toc103528743)

[**Figure S3B. The number of participants with extreme values for each IDP for PGS analysis.** 3](#_Toc103528744)

[2. Supplementary result 4](#_Toc103528745)

[**Figure S4. Impact of (categorical) demographic factors on wellbeing-index phenotype.** 4](#_Toc103528746)

[**Figure S5. Brain regions that were significantly associated with the wellbeing-index phenotype and/or wellbeing-PGS in the mediation analysis sample (n=19,461)………………………………………………………………** 5](#_Toc103528748)

[**Figure S6. Sex-stratified IDP-wellbeing association.** 6](#_Toc103528750)

[Supplementary references 7](#_Toc103528751)

# 1. Supplementary methods

## 1.1. Derivation of the Wellbeing-index

The wellbeing-index was calculated for participants with neuroimaging data available, using the same method we validated in our previous study.(1) The method is summarised below in detail.

Six items from the questionnaire provided at the first neuroimaging visit were identified that related to wellbeing concept. These items were a general question on happiness;

1) "In general, how happy are you?",

and five questions on different domains of life satisfaction – "In general, how satisfied are you with your…"

2) …family relationships,

3) …friendships,

4)…financial situation,

5)…health,

6)…work that you do;

These questions [Data-Fields 4526, 4559, 4570, 4581, 4548, 4537 respectively] were rated on a 6-point Likert scale (e.g., from 'extremely happy' to 'extremely unhappy'). Participants with "Do not know" or "Prefer not to answer" were excluded. As 43.5% of the sample had missing data for work satisfaction, we excluded work satisfaction from the factor analysis.

The single item that related to psychological wellbeing was evaluated at follow-up assessment in the mental health survey for participants undertaking the neuroimaging study component. Due to the significant time-lag between baseline and follow-up assessments and the variability in follow-up period, this psychological wellbeing item was excluded from the present study.

A principal component analysis (PCA) in SPSS (v.24) was performed on the five wellbeing related items to derive the wellbeing-index score. Only the first principal component (PC1) had an Eigenvalue greater than 1 (Eigenvalue = 2.414), which was used as the wellbeing-index. The PC1 explained 48.3% of the variance in the questions and had a correlation of 0.78,0.75,0.73,0.61 and, 0.60 with happiness, family satisfaction, friendship satisfaction, financial satisfaction, and health satisfaction, respectively. The scree plot from the PCA analysis and the distribution of the wellbeing-index score in the present sample is presented in Figure S1.


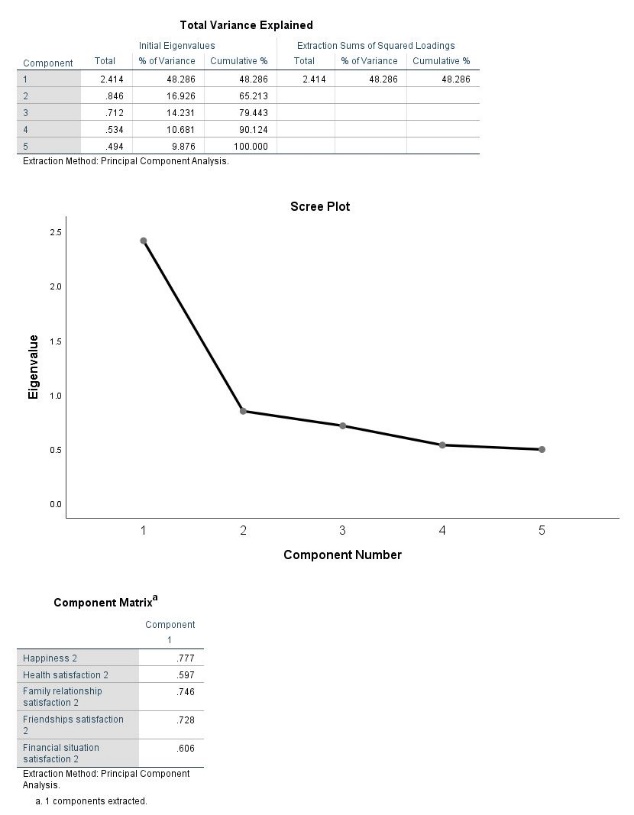

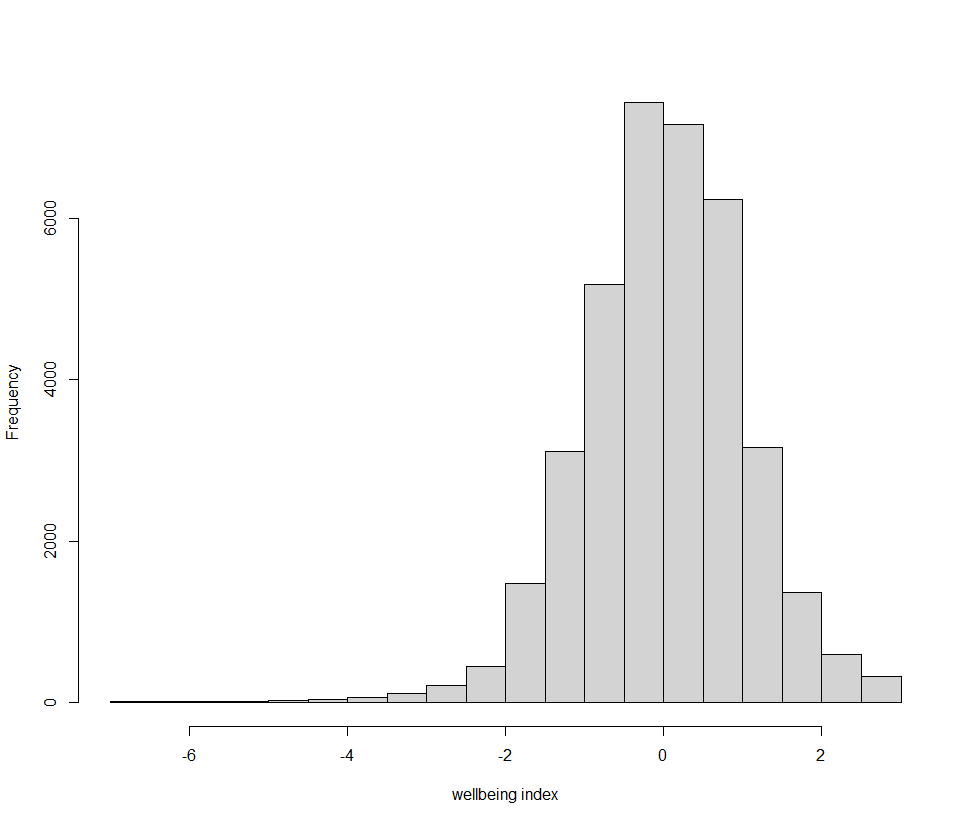


A

B

### **Figure S1. Scree plot and distribution of the wellbeing-index score.** A) The scree plot from the principal component analysis (PCA) and, B) Distribution of the wellbeing-index in the sample.

## 1.2. Sample selection

Participants with brain imaging data available

**N = 42,809**

Exclude participants with confounding diseases

**N = 40,993**

Exclude participants with extreme ICV (M±3SD)

**N = 40,855**

Exclude participants with missing covariates

**N = 40,087**

Participants with *wellbeing-index* available

**N = 38,982**

Participants with *genotype data* available

**N = 36,095**

Participants with *wellbeing*-*PGS* available

[after exclusions based on QC, ethnicity & relatives (kinship-IBD>0.04) in discovery GWAS]

**N = 19,987**

**Figure S2. The sample selection workflow.** Confounding diseases [UKB Data-Fields 20002 and 20001] that resulted in participant exclusion included brain haemorrhage, brain abscess, cerebral aneurysm, cerebral palsy, chronic/degenerative neurological problem, dementia/Alzheimer's/ cognitive impairment, encephalitis, head injury, infection of nervous system, ischaemic stroke, epilepsy, meningioma, meningitis, motor neuron disease, multiple sclerosis, neurological injury/trauma, benign neuroma, other neurological problems, Parkinson's disease, spina bifida, stroke, subarachnoid haemorrhage, subdural haemorrhage, transient ischaemic attack, peripheral nerve/autonomic nerve cancer, meningeal cancer/malignant meningioma, brain cancer/primary malignant brain tumour. Covariates that were required for inclusion were age, sex, head position (X, Y, Z) in scanner, assessment centre, education level, ethnicity, Townsend deprivation index, smoking status, alcohol intake frequency, and body mass index (BMI). Participants included in wellbeing-PGS analysis were of ‘British-White’ background, unrelated to participants in the discovery GWAS (kinship-IBD>0.04) and passed all other QC measures, and included n=525 participants with genotype data who were absent from the phenotype sample.

## 1.3. Covariates

Head positions in the MRI scanner that were included as covariates, are scanner lateral (X) [Data-Field:25756], transverse (Y) [Data-Field:25757], and longitudinal (Z) [Data-Field:25758] co-ordinates of the centre of the brain mask within the scanner.

Education was coded as a binary variable of either college or no college degree. We used Data-Field:6138 (Qualifications) and coded "*College or University degree"* as college degree and all other answers as no college degree. *"Prefer not to answer"* was coded as missing.

For ethnicity, Data-Field:21000 (ethnic background) was used. People with “White” ethnic backgrounds (coding 1,1001,1002, and 1003) were coded as white, and all other groups were coded as non-white. "Do not know" and "Prefer not to answer" were coded as missing. In the final samples, all participants with “White” ethnic background were “British White”.

Townsend deprivation index at recruitment [Data-Field:189] is a score corresponding to the output area in which the participant postcode is located.

Smoking status [Data-Field:20116] summarises the current/past smoking status of the participant in three categories of "never", "previous" or "current". "Prefer not to answer" was coded as missing.

Alcohol intake frequency [Data-Field:1558] is the answer to "About how often do you drink alcohol?" with six categories: "Daily or almost daily", "Three or four times a week", "Once or twice a week", "One to three times a month", "Special occasions only", and "Never". "Prefer not to answer" was coded as missing.

Body mass index (BMI) [Data-Field:21001] is calculated using a person's height and weight with the formula: BMI = kg/m^2^. We divided the BMI values into four categories: underweight (BMI<18.5), normal (18.5≤BMI<25), overweight (25≤BMI<30) and obese (BMI>30). The categorical variable was used as the covariate.

All of these variables were collected at the first imaging visit.

###
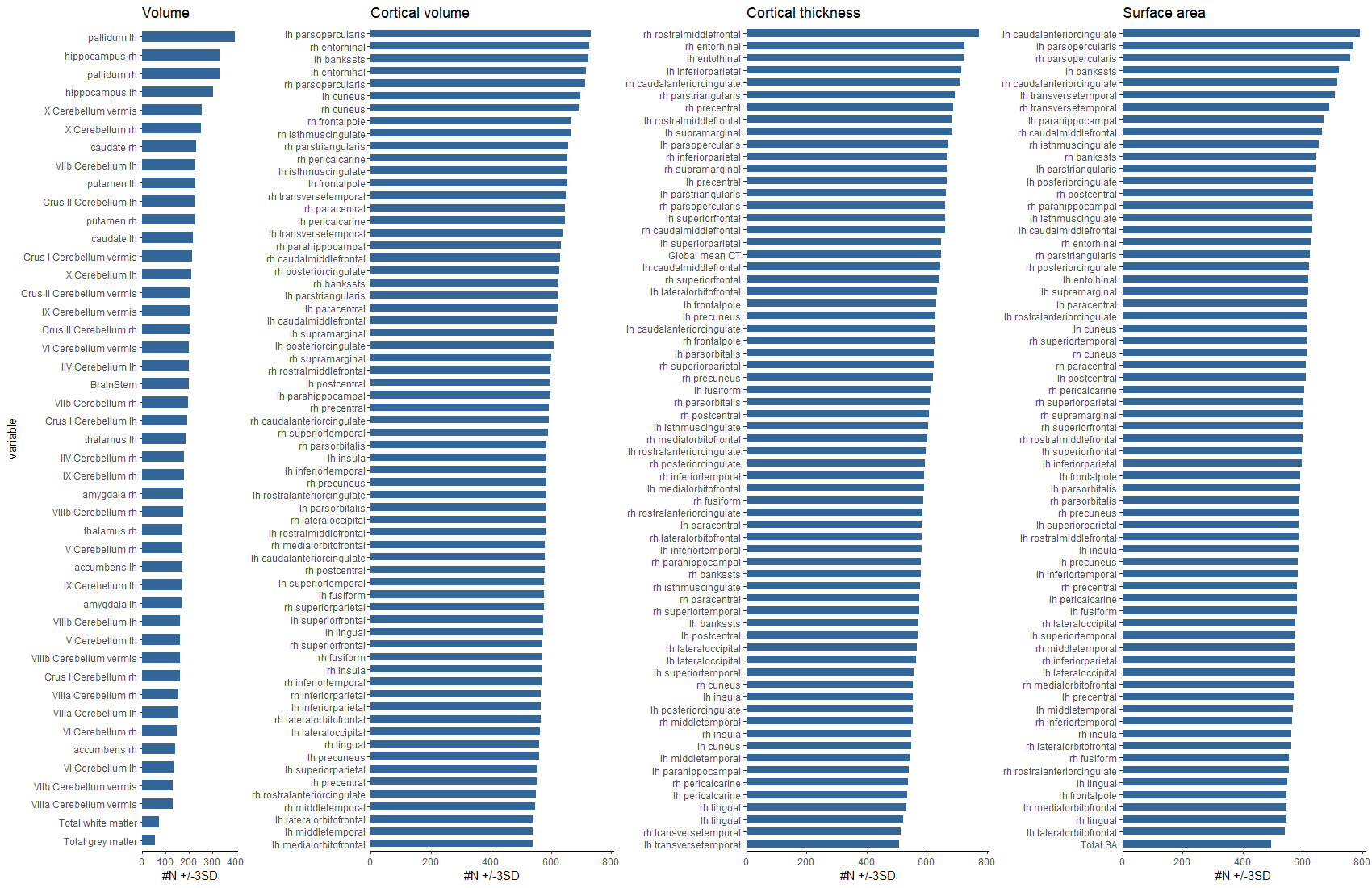
**Figure S3A. The number of participants with extreme values for each IDP in wellbeing-index phenotype analysis.** Values outside ±3 standard deviations from the mean were excluded from each corresponding analysis. *Abbreviations:* lh, left hemisphere; rh, right hemisphere.

###
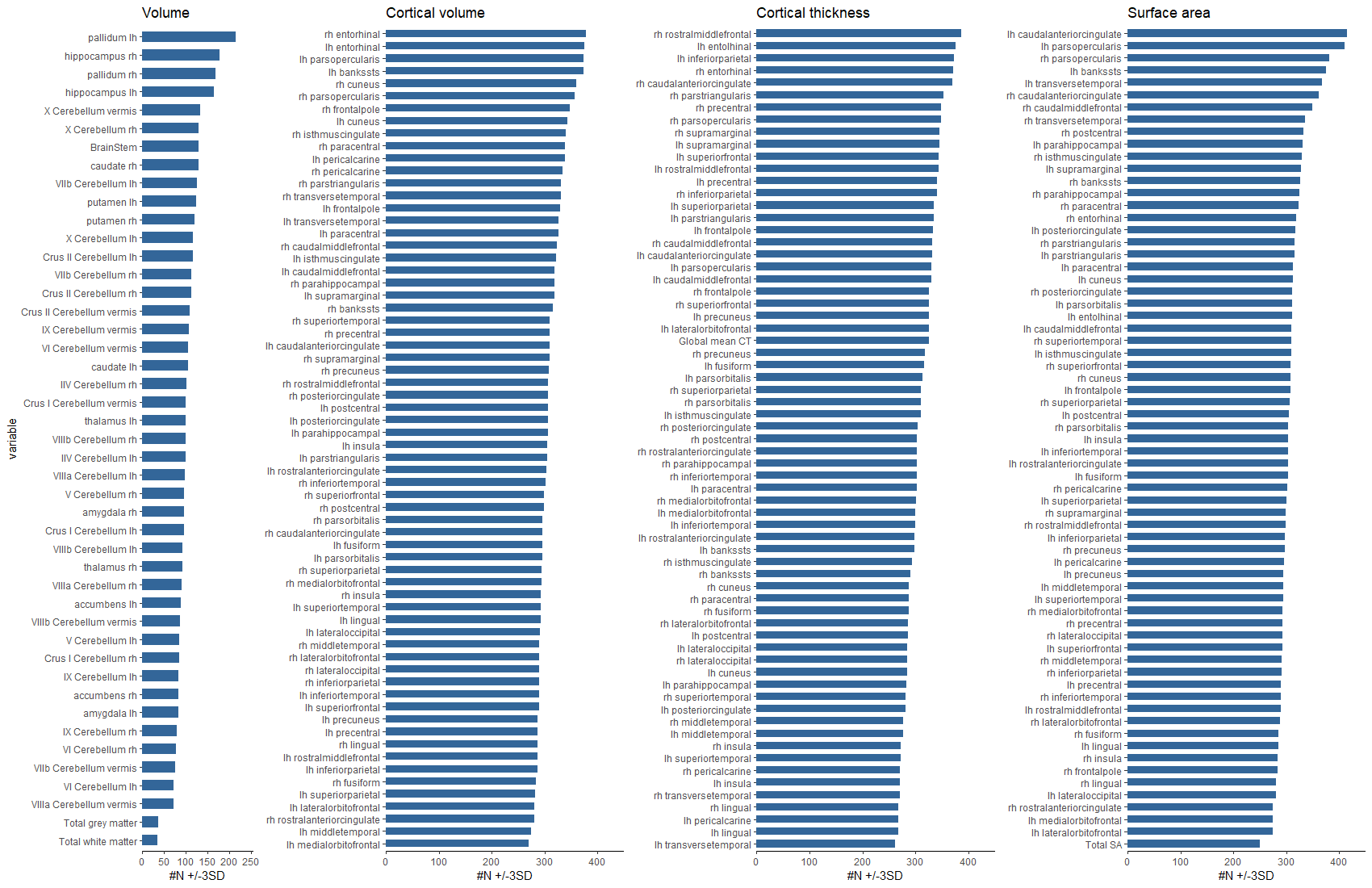
**Figure S3B. The number of participants with extreme values for each IDP for PGS analysis.** Values outside ±3 standard deviations from the mean were excluded from each corresponding analysis. *Abbreviations:* lh, left hemisphere; rh, right hemisphere.

#
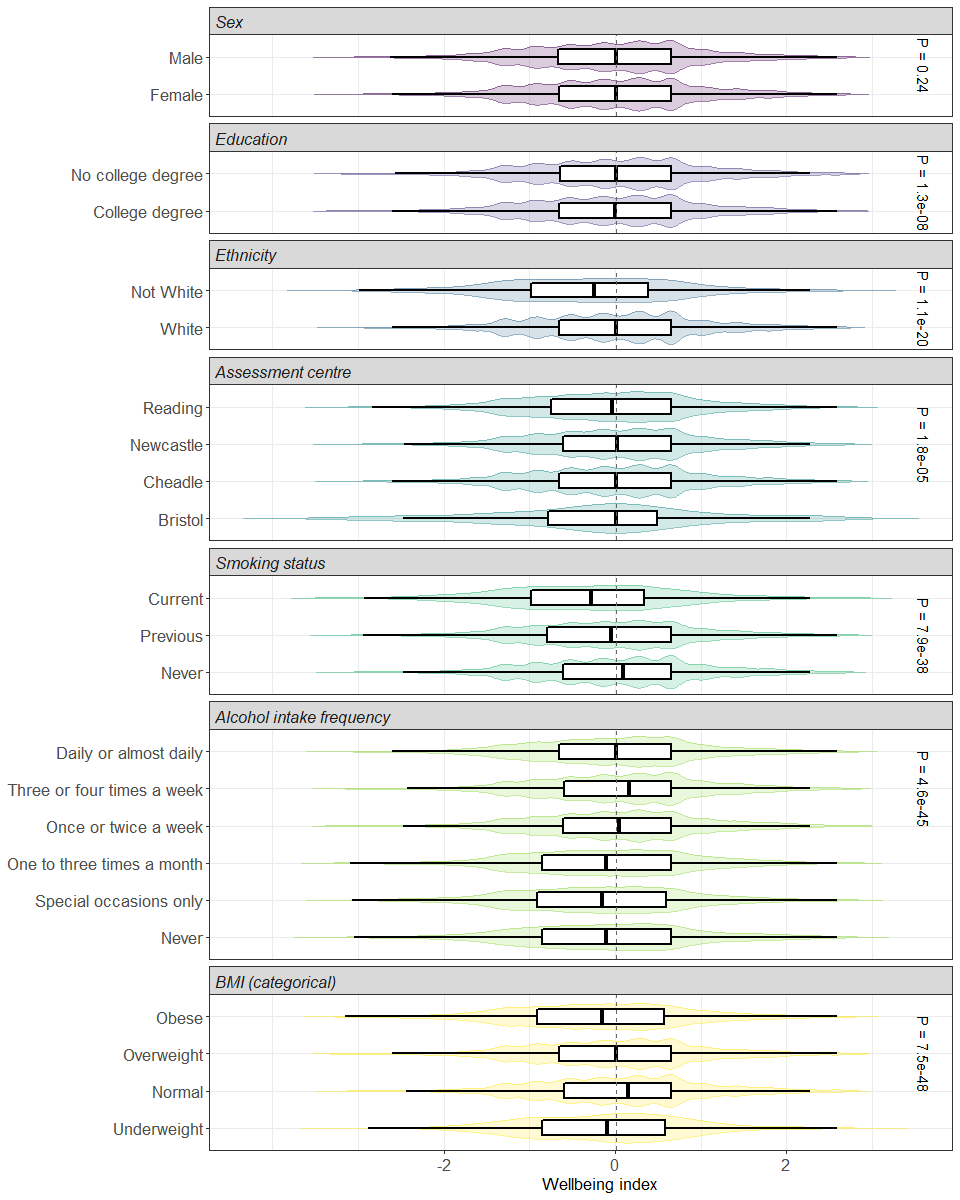
2. Supplementary result

### **Figure S4. Impact of (categorical) demographic factors on wellbeing-index phenotype.** The line in the middle of the boxplots shows the median of each group and the lower and upper hinges correspond to the first and third quartiles (the 25th and 75th percentiles). The upper/lower whiskers extend from the hinge to the largest/smallest value no further than (1.5×Inter-quartile range) from the hinge. The violin plots that appear behind the boxplots show the distribution of wellbeing-index phenotype in each group. From these variables, only sex and assessment centre. *Abbreviations:* BMI, body mass index; P, p-value.

### **
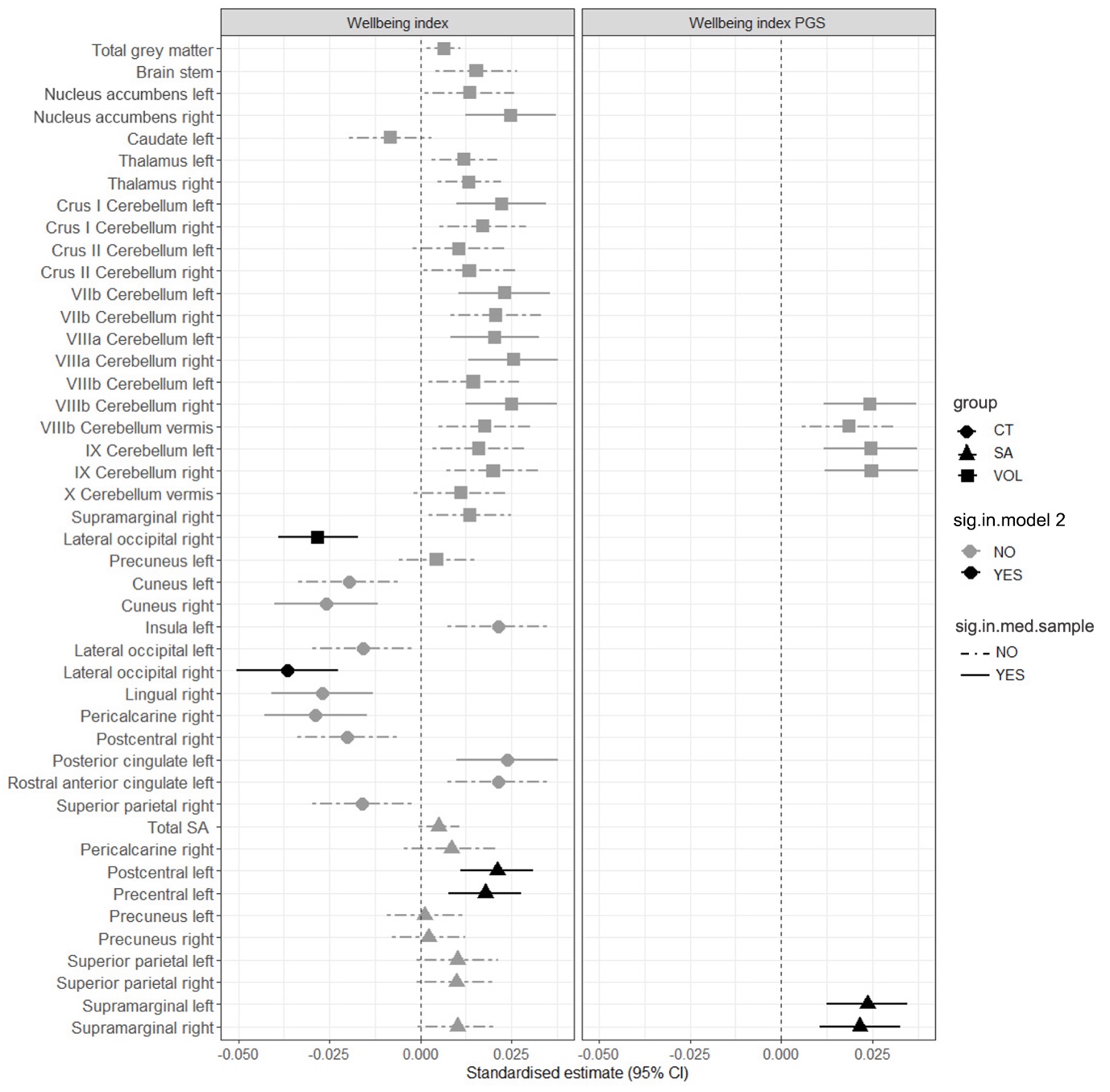
**

### **Figure S5. Brain regions that were significantly associated with the wellbeing-index phenotype and/or wellbeing-PGS in the mediation analysis sample (n=19,461).** The x-axis shows the standardised estimates (β) with 95% confidence interval from model 1 (included covariates: age, age^2^, sex, ICV, assessment centre, and scanner x-, y- and z-axis positions; plus genotype array and the first ten principal components of the genetic data for PGS association analysis). Image-derived phenotypes (IDP)s derived from estimates of volume, surface area, and thickness are denoted by squares, triangles, and circles, respectively. The IDPs that remained significant in model 2 (inclusion of additional covariates: education, ethnicity, Townsend deprivation index, smoking status, alcohol intake frequency, and body mass index) are shown in black (effect sizes relate to model 1). The dotted confidence interval lines indicate associations that were significant in the main analysis sample but were not significant (P_FDR_>0.016) in the mediation (smaller) sample.

###
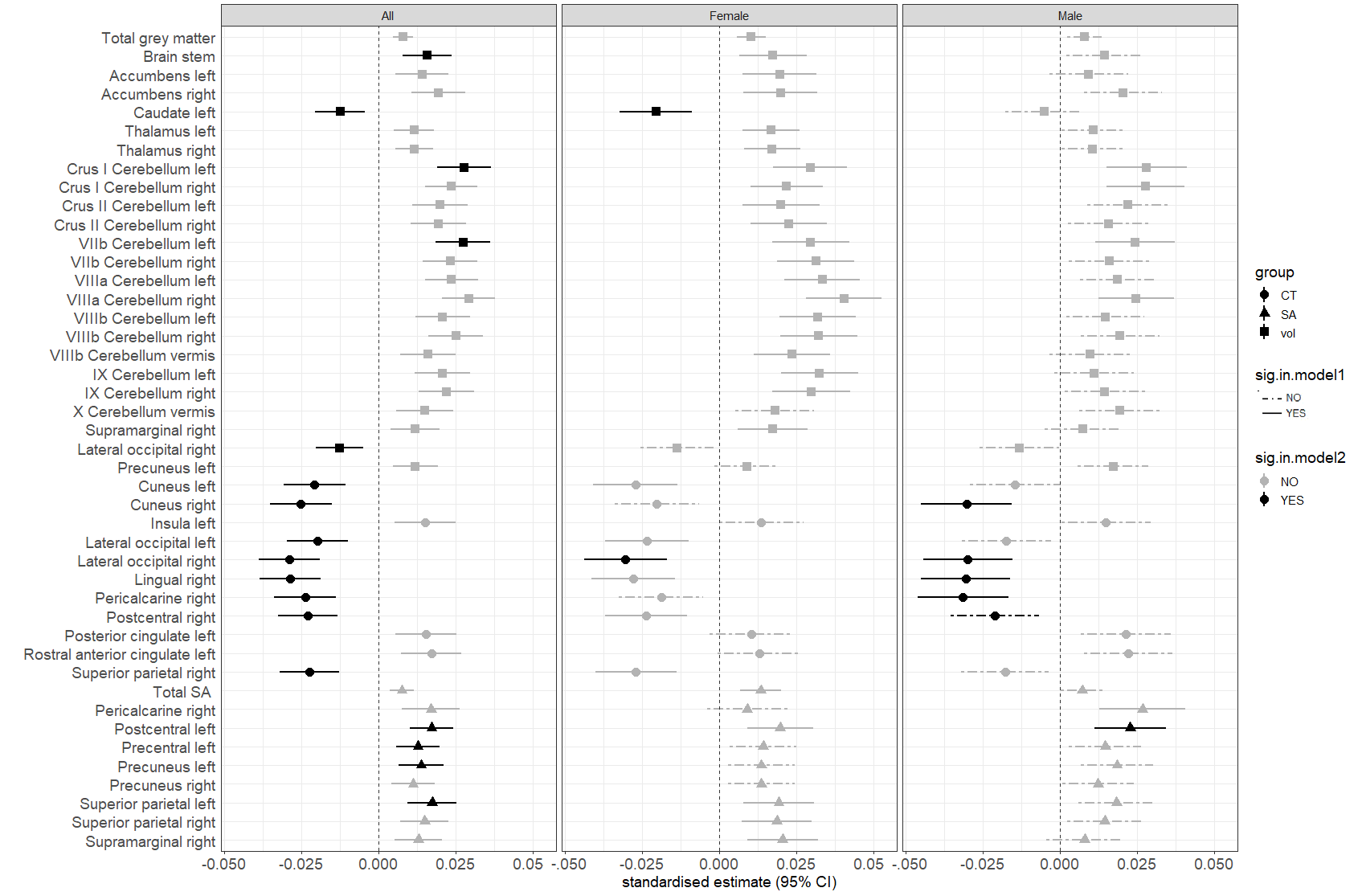


### **Figure S6. Sex-stratified IDP-wellbeing association. Only IDPs with significant association in the whole sample (all; N= 38,982) were separately analysed for association in female (N= 20,776), and male (N= 18,206) subsets.** The x-axis shows the standardised estimates (β) with 95% confidence interval from model 1 (included covariates: age, age^2^, sex, ICV, assessment centre, and scanner x-, y- and z-axis positions). Image-derived phenotypes (IDP)s derived from estimates of volume, surface area, and thickness are denoted by squares, triangles, and circles, respectively. The IDPs that remained significant in model 2 (including model 1 covariates plus additional covariates: education, ethnicity, Townsend deprivation index, smoking status, alcohol intake frequency, and body mass index) are shown in black (effect sizes relate to model 1). The dotted confidence interval lines indicate associations that were significant in the whole sample but were not significant (P_FDR_>0.016) in female or male subsets.

# Supplementary references

1. Jamshidi J, Schofield P, Gatt J, Fullerton J (2022): Phenotypic and genetic analysis of a wellbeing factor score in the UK Biobank and the impact of childhood maltreatment and psychiatric illness. *Transl Psychiatry*. In Press.
